# Genomic Surveillance and Mutation Analysis of SARS-CoV-2 Variants among Patients in Saudi Arabia

**DOI:** 10.3390/microorganisms12030467

**Published:** 2024-02-26

**Authors:** Feda A. Alsuwairi, Asma N. Alsaleh, Dalia A. Obeid, Ahmed A. Al-Qahtani, Reem S. Almaghrabi, Basma M. Alahideb, Maha A. AlAbdulkareem, Madain S. Alsanea, Layla A. Alharbi, Sahar I. Althawadi, Sara A. Altamimi, Abeer N. Alshukairi, Fatimah S. Alhamlan

**Affiliations:** 1 Department of Infection and Immunity, King Faisal Specialist Hospital and Research Center, Riyadh 11211, Saudi Arabia; feadh.sa@gmail.com (F.A.A.); obeiddx@gmail.com (D.A.O.); aqahtani@kfshrc.edu.sa (A.A.A.-Q.); balahideb@kfshrc.edu.sa (B.M.A.); moalabdulkareem@kfshrc.edu.sa (M.A.A.); maalsanea@kfshrc.edu.sa (M.S.A.); lalharbi8@kfshrc.edu.sa (L.A.A.); 2 Botany and Microbiology Department, College of Science, King Saud University, P.O. Box 2460, Riyadh 11451, Saudi Arabia; asmalsaleh@ksu.edu.sa; 3 Organ Transplant Center of Excellence, King Faisal Specialist Hospital and Research Center, Riyadh 11564, Saudi Arabia; ramaghrabi@kfshrc.edu.sa; 4 College of Medicine, Alfaisal University, Riyadh 11533, Saudi Arabia; aalshukiri@kfshrc.edu.sa; 5 Department of Pathology and Laboratory Medicine, King Faisal Specialist Hospital and Research Center, Riyadh 11211, Saudi Arabia; sthawadi@kfshrc.edu.sa (S.I.A.); saltamimi98@kfshrc.edu.sa (S.A.A.); 6 Department of Medicine, King Faisal Specialist Hospital and Research Centre, Jeddah 23433, Saudi Arabia

**Keywords:** COVID-19, SARS-CoV-2, genome, variants, amino acid, mutations, spike (S), envelope (E), nucleocapsid (N), membrane (M)

## Abstract

The genome of severe acute respiratory coronavirus-2 (SARS-CoV-2), the virus responsible for coronavirus disease 2019 (COVID-19), has undergone a rapid evolution, resulting in the emergence of multiple SARS-CoV-2 variants with amino acid changes. This study aimed to sequence the whole genome of SARS-CoV-2 and detect the variants present in specimens from Saudi Arabia. Furthermore, we sought to analyze and characterize the amino acid changes in the various proteins of the identified SARS-CoV-2 variants. A total of 1161 samples from patients diagnosed with COVID-19 in Saudi Arabia, between 1 April 2021 and 31 July 2023, were analyzed. Whole genome sequencing was employed for variant identification and mutation analysis. The statistical analysis was performed using the Statistical Analytical Software SAS, version 9.4, and GraphPad, version 9.0. This study identified twenty-three variants and subvariants of SARS-CoV-2 within the population, with the Omicron BA.1 (21K) variant (37.0%) and the Delta (21J) variant (12%) being the most frequently detected. Notably, the Omicron subvariants exhibited a higher mean mutation rate. Amino acid mutations were observed in twelve proteins. Among these, the spike (S), ORF1a, nucleocapsid (N), and ORF1b proteins showed a higher frequency of amino acid mutations compared to other the viral proteins. The S protein exhibited the highest incidence of amino acid mutations (47.6%). Conversely, the ORF3a, ORF8, ORF7a, ORF6, and ORF7b proteins appeared more conserved, demonstrating the lowest percentage and frequency of amino acid mutations. The investigation of structural protein regions revealed the N-terminal S1 subunit of the S protein to frequently harbor mutations, while the N-terminal domain of the envelope (E) protein displayed the lowest mutation frequency. This study provides insights into the variants and genetic diversity of SARS-CoV-2, underscoring the need for further research to comprehend its genome evolution and the occurrence of mutations. These findings are pertinent to the development of testing approaches, therapeutics, and vaccine strategies.

## 1. Introduction

Severe acute respiratory syndrome coronavirus-2 (SARS-CoV-2) is an etiological agent of the respiratory disease coronavirus disease 2019 (COVID-19). The virus was first reported in the live animal and seafood market in the Wuhan province of China in December 2019 [1]. Since its emergence, along the course of its spread and infections caused, rapid evolutionary events in the RNA genome of the virus have been reported, through the contribution of molecular mechanisms of genetic diversity, such as replication errors and host-dependent RNA editing or recombination [2,3,4]. The rapid evolution of the virus has resulted in several SARS-CoV-2 variants or lineages that have emerged in different geographic regions. These variants have been reported to have accumulated several amino acid changes, and have appeared with distinguished phenotypes that may influence the epidemiological and clinical characteristics of COVID-19. For example, they can enhance the virus transmissibility or infectivity, and in some cases may result in severe disease or confer immune evasion to the virus [2,5].

The enveloped virus SARS-CoV-2 comprises a linear, non-segmented, positive-sense, single-stranded RNA genome (+ssRNA) with a 5′-cap and 3′- poly (A) tail [6,7]. The virus has a large genome, with 29,903 bp in nucleotide length, which translates into 9860 amino acids [8]. The genes of SARS-CoV-2 are organized into 14 known open-reading frames (ORFs) [9]. The overlapping ORFs are as follows: ORF1ab is the largest SARS-CoV-2 gene, accounting for about two-thirds of the genome. The two ORFs are translated into two large polyproteins: (1) the ORF1a gene is translated into polyprotein pp1a, and (2) the ORF1b gene is translated into polyprotein pp1ab [10,11]. These polyproteins are proteolyzed by virus proteases, resulting in the production of 16 non-structural proteins (NSP1-NSP16) [12]. The remaining ORFs are encoded at the 3′ end and form about one-third of the genome. These ORFs encode four structural proteins, namely the spike (S), the envelope (E), the nucleocapsid (N), and the membrane (M) [13]. These structural proteins play a crucial role in the infectivity and pathogenesis of the virus. Each of these four proteins has specific functional domains. The S protein, for instance, consists of two subunits, N-terminal S1 and C-terminal S2, which are involved in receptor binding and membrane fusion to facilitate viral entry into the host cell [14]. The N protein comprises an N-terminal domain (NTD), a serine and arginine (SR)-rich central linker region (LKR), and a C-terminal domain (CTD), which are essential for assembling and packaging the RNA into the form of a ribonucleocapsid protein (RNP) complex [15]. The E protein contains three domains: a short hydrophilic N-terminal domain, a transmembrane hydrophobic domain, and a C-terminal tail that promotes virion formation and pathogenicity, while the M protein comprises an N-terminal ectodomain, a triple-membrane-spanning domain, and a long C-terminal endodomain, which are involved in virion assembly [16,17]. The structural protein ORFs are interspersed with ORFs that encode accessory genes, some of which are overlapped ORFs. These are ORF3a, ORF3b, ORF6, ORF7a, ORF7b, ORF8, ORF9b, ORF9c, and ORF10 [13].

Random or continuous mutation can occur in the viral genes or proteins. However, the tendency of a single SARS-CoV-2 gene/protein to mutate can vary, though [18]. Thus, comparing the occurrence of mutations in the genes/proteins can help in identifying variable or conserved regions [19]. The variable regions in the proteins of viruses are important because they play a crucial role in the virus’s ability to evade detection and infect the host cells. These regions are responsible for the genetic diversity of viral proteins of different variants. They can influence virus neutralization by antibodies, viral pathogenicity, and virulence [20,21]. Conserved regions can serve as stable viral targets and potential antigenic sites for the development of vaccines, effective therapeutics against variants of the virus, or sensitive diagnostics tools [19,22]. Identifying the conserved regions in SARS-CoV-2 variants and other coronaviruses can assist in determining a target for developing an effective universal vaccine against these viruses. Accordingly, the efficacy of the vaccines against SARS-CoV-2 infections decreased over time as mutations continuously occurred in the SARS-CoV-2 genome and multiple variants were detected. These efforts are still necessary to work on developing a universal vaccine against the virus [18,22]. Here, our objective is to employ a next-generation sequencing approach to sequence the complete genome of SARS-CoV-2 and identify the variants present in patient specimens. Additionally, we aim to conduct a comprehensive analysis and characterization of the mutations occurring in the different proteins of the identified SARS-CoV-2 variants. Through this research, our aim is to gain a deeper understanding of the genetic diversity exhibited by various SARS-CoV-2 variants.

## 2. Methods

### 2.1. Ethical Approval

Ethical approval was obtained from the Research Ethics Committee (REC) of King Faisal Specialist Hospital and Research Centre, Saudi Arabia (KFSH&RC: RAC #2200009). Given the retrospective nature of this study and the utilization of anonymized data, the requirement for obtaining informed consent from patients was waived. The research was conducted in accordance with the principles outlined in the Declaration of Helsinki.

### 2.2. Clinical Specimen and Data Collection

A total of 1161 nasopharyngeal swab (NPS) specimens, confirmed positive for SARS-CoV-2 through a polymerase chain reaction (PCR), were collected from the Microbiology Section of the Pathology and Laboratory Medicine Department at KFSHRC from three different cities in Saudi Arabia [Riyadh, Jeddah, and Madinah]. These specimens were deidentified, coded, and obtained in viral transport media. The sample collection spanned from 1 April 2021 to 31 July 2023.

### 2.3. Whole Genome Sequencing

Unless otherwise noted, all the whole genome sequencing equipment and kits were purchased from Thermo Fisher Scientific, Waltham, MA, USA. The total viral RNA was extracted from 200 μL of commercially available viral transport media that was stored at -80 °C by utilizing the MagMAX^TM^ Viral/Pathogen II Nucleic Acid Isolation Kit. Using a Nanodrop system, the RNA integrity was assessed; a 260/280 ratio of around 2.00 was considered acceptable. The detection of the viral RNA and the estimation of the viral load were conducted using a TaqPath™ COVID-19 CE-IVD RT-PCR Kit that specifically targets the genes of SARS-CoV-2: the nucleocapsid (N), spike(S), and open reading frame 1ab (ORF1ab).

The extracts that were positive by real-time PCR were converted into cDNA using an Invitrogen SuperScript™ IV VILO™ Master Mix kit. The assays were conducted according to the manufacturer’s instructions. The cDNA was used to prepare libraries with the Ion AmpliSeq™ SARS-CoV-2 Research Panel. The preparation of the libraries included (1) amplification of the targets, (2) partial digestion of the amplicons, and (3) ligation of the adapters to the amplicons. The amplification of the targets was conducted for each sample through two separate amplification reactions, with primer pool 1 used in one reaction and primer pool 2 used in the other. The amplification reactions were then combined. The amplified cDNA target was then partially digested. The products were ligated with unique barcode adapters using Ion Xpress™ Barcode Adapters 1-96 Kits. Each library was purified using 45 µL (i.e., 1.5× sample volume) of Agencourt™ AMPure™ XP Reagent (Beckman Coulter, Brea, CA, USA). The library preparation was performed according to the Ion AmpliSeq™ Library Kit Plus User Guide (MAN0006735) and following the Ion AmpliSeq™ RNA Libraries protocol. All the reactions were performed using a VeritiTM 96-well Thermal Cycler. All the barcoded libraries were quantified using an Ion Library TaqMan Quantitation Kit, normalized from the original libraries using nuclease-free water at 28–33 pM for the Ion 520–530 chip or at 50 pM for the Ion 540 chip, and were then pooled into equal volumes based on the selected Ion chip capacity, prior to undergoing automated template preparation. The template preparation included emulsion PCR and then the immobilization of each DNA fragment on Ion Sphere™ Particles. These cloned DNA fragments were loaded into the wells of an electronic semiconductor chip (Ion 520™ Chip, Ion 530™ Chip, or Ion 540™ Chip). The automatic template preparation was performed using an Ion Chef™ Instrument with an Ion 510™ & Ion 520™ & Ion 530™ Kit-Chef or with an Ion 540™ Kit-Chef. Whole genome sequencing was performed with the Ion GeneStudio™ S5 System using Ion S5 sequencing solutions from the Ion 510™ & Ion 520™ & Ion 530™ Kit–Chef or the Ion 540™ Kit-Chef.

### 2.4. Data Analysis and Phylogenetic Analysis

Torrent Suite™ Software version 5.12 (Thermo Fisher Scientific, Waltham, MA, USA) was used to analyze the sequencing data. The sequences were aligned with the reference genome of the Wuhan-Hu-1 reference genome (accession number MN908947.3). The following plugins were used in the analysis: AssemblerTrinity (v1.2.1.0), coverageAnalysis (v5.10.0.3), variantCaller (v5.10.1.19), COVID19AnnotateSnpEff (v1.2.1.0), and IRMAreport (v1.2.1.0). AssemblerTrinity (v1.2.1.0) was used to perform a de novo report of the assembled contigs into a FASTA file format. CoverageAnalysis (v5.10.0.3) contains matrices for analyzing the quality control of each sequence. VariantCaller (v5.10.1.19) was used to call Single-nucleotide and indel variants. Variant call files were analyzed with the COVID19AnnotateSnpEff plugin (v1.2.1.0) to predict the effects of mutations on the nucleic acid level and on the amino acid level. IRMAreport (v1.2.1.0) was used to generate consensus sequences for the samples into a FASTA file format. For the analyzed consensus sequences, the Nextclade web tool v2.14.1 “https://nextstrain.org (accessed on 1 September 2023)” was used to align the sequences from our population and compare them with the Wuhan-Hu-1 sequence to check for deficiencies in the sequence that could have occurred during sequencing or assembly. The sequences issues were resolved by removing gaps and contaminants using DNASTAR Lasergene Software package (DNASTAR; Madison, WI, USA). Further, the mutations per sample were exported from the Nextclade web tool into an Excel format file and then calculated and analyzed. A Nextclade tree was also constructed using their online tool “https://nextstrain.org (accessed on 10 November 2023)”. The tree was then downloaded into JSON format and visualized using the Auspice v2.34.0 online tool “https://auspice.us/ (accessed on 11 November 2023)”.

### 2.5. Data Statistical Analysis

All the collected data were analyzed using SAS (version 9.4) software and GraphPad Prism (version 9.0). Inferential and descriptive statistical analyses were performed to evaluate the characteristics of the patients based on their genomic data.

## 3. Results

### 3.1. Demographic and Clinical Characteristics of this Study’s Patients

A total of 1161 NPS samples were obtained and analyzed. The majority of samples, 85.1% (*n* = 988), were from Riyadh, followed closely by Jeddah at 7.9% (*n* = 92) and Madinah at 7.0% (*n =* 81). Among the patients, the majority were Saudi nationals, 73.8% (*n* = 807), and nonsmokers, 92.8% (*n =* 999). The sample comprised 55.7% females (*n* = 647), which was a higher percentage compared to the 44.3% of males (*n* = 514). The mean age of the patients was 39.4 years (SD = 18.2), with the youngest patient being three weeks old and the eldest being 102 years old.

Regarding vaccination status, the majority of patients, 92.3% (*n* = 674), had been vaccinated before the infection, while 7.7% (*n* = 56) were unvaccinated. Among the vaccinated individuals, the most common vaccine received was an mRNA vaccine (Pfizer BioNTech or Moderna), 49.5% (*n* = 313), followed by an Adenoviral Vector-Based Vaccine (AstraZeneca), 23.2% (*n* = 147). Approximately 27.3% of the patients (*n* = 173) received vaccines from more than one company. In terms of vaccine doses, the majority of the patients, 45.3% (*n* = 282), received booster doses, while 25.4% (*n* = 158) received only the first dose and 29.4% (*n* = 183) received a second dose.

By the end of the data collection stage of our study, which was 31 July 2023, the following observations had been made: 79.5% (*n* = 917) of the patients had recovered without requiring hospitalization, while 20.5% (*n* = 237) were hospitalized. Among the hospitalized patients, 11.0% (*n* = 125) required a short period of hospitalization lasting up to 20 days, 8.4% (*n* = 95) required longer hospital stays, and the hospitalization duration for 27 patients was unknown. Unfortunately, 4.1% (*n* = 47) of the patients succumbed to the disease.

In terms of comorbidities, 52.7% (*n* = 596) of the patients did not have any underlying health conditions, while 47.3% (*n* = 535) did. Among those with comorbidities, 24.9% (*n* = 281) were immunocompromised individuals, including 8.3% (*n* = 95) who had received organ transplants. Additionally, 15.9% (*n* = 180) of the patients had diabetes, and 23.0% (*n* = 261) had hypertension.

The majority of the patients, 96.4% (*n* = 993), experienced symptoms related to COVID-19, while a smaller portion, 3.6% (*n* = 37), were asymptomatic. Among the symptomatic patients, 11.7% (*n* = 134) had severe infections, while the majority, 84.7% (*n* = 978), had mild-to-moderate illness severity. Furthermore, 9.7% (*n* = 110) of the patients required admission to an intensive care unit (ICU).

### 3.2. Distribution of SARS-CoV-2 Variants among the Genomic Sequences of the Patients

The detected SARS-CoV-2 variants were identified using the nomenclature systems of the World Health Organization (WHO), PANGO Lineage, and Nextstrain [23,24,25]. A total of twenty-three different variants and subvariants were identified, with the most frequent variant being Omicron BA.1 (21K), accounting for 37.0% of the cases. This was followed by Delta (21J) (12%), Omicron XBB.1.9 (23D) (11.5%), Omicron BA.5 (22B) (8.1%), Omicron XBB (22F) (6.9%), and Delta (21I) (2.9%).

The prevalence of these variants varied during different time periods of this study. The Alpha (20I) variant was dominant from April 2021 to late May 2021, with the Beta (20H) variant also circulating during the same period. After May 2021, the Alpha and Beta variants declined, and the Delta (21J) variant became the predominant variant. Delta peaked in June 2021 and continued until late November 2021. From April 2021 to November 2021, other variants, including A.29 (19B), C.36.3 (20D), Eta (21D), and Kappa (21B), were noted at low frequencies and were only present for short periods.

**Figure 1:**
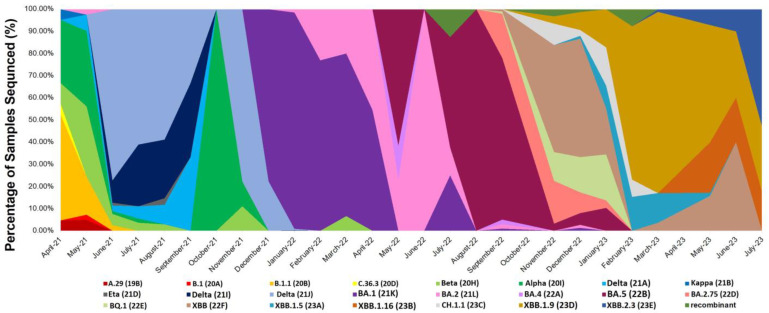
The distribution of SARS-CoV-2 detected variants and subvariants identified through a sequence analysis from April 2021 to July 2023 is shown. The bar graph displays the percentage of samples detected with variants, showcasing the distribution of the variants in specific months. Each color represents a specific variant and the period in which the variant was detected. During the first month of analysis, the dominant variant was the Alpha variant, observed from April 2021 to late May 2021. In June 2021, Delta dominated and continued to until late November 2021. Beginning in December 2021, Omicron subvariants were observed in the cohort.

**Figure 2:**
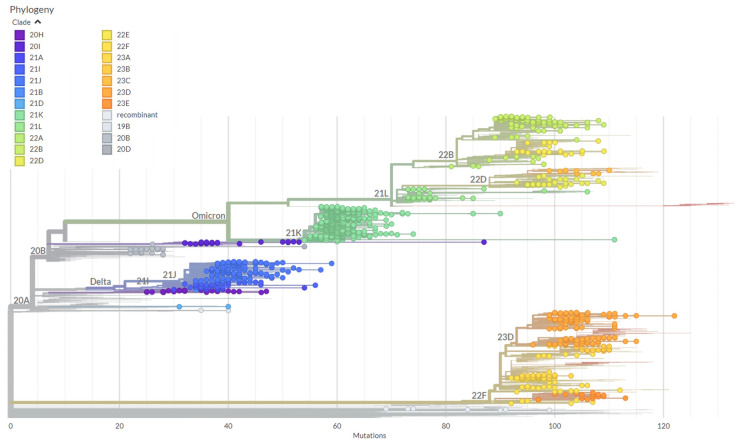
A rectangular phylogenetic tree of SARS-CoV-2, spanning from 1 April 2021 to 31 July 2023, was constructed. The reference tree used in this study was Wuhan-Hu-1/2019 (MN908947), and it was utilized alongside the sequences analyzed. The distribution of different clades is depicted through specific-colored dots, which are assigned Nextstrain and WHO clade names. The lengths of the tree branches represent the number of mutations in the clades. The construction of the phylogenetic tree was achieved using the Nextstrain pipeline.

Different subvariants of the Omicron variant were observed in the cohort. The first subvariant, BA.1, emerged and replaced the Delta variant, dominating from December 2021 to April 2022. During this time, BA.2 (21L) was also found to be circulating. In May 2022, BA.5 replaced BA.1 and became the most abundant variant from September 2022 to October 2022. Alongside BA.5, BA.4 (22A) and BA.2.75 (22D) were also circulating. As of November 2022, the frequencies of BA.5 and BA.4 decreased. Several other Omicron subvariants, including BQ.1 (22E), XBB (22F), and CH.1.1 (23C), appeared. Among them, XBB (22F) was the most common and remained dominant until January 2023. The frequency of XBB (22F) decreased starting from February 2023, while the frequencies of both XBB.1.5 (23A) and XBB.1.9 increased. From February 2023 to June 2023, XBB.1.9 became the most common subvariant. In May 2023, XBB.2.3 (23E) was observed and remained predominant until the end of this study on 31 July 2023. Additionally, four unassigned SARS-CoV-2 isolates were observed and referred to as recombinant. The Nextstrain phylogenetic tree of the sequences confirmed the identities of the respective SARS-CoV-2 variants and sub-variants, resulting in 23 distinct clades based on the Nextclade classification. Table 1 shows all the variants detected in the cohort, and Figure 1 illustrates the distribution of the variants and subvariants over the different time periods. The phylogenetic tree depicting the relationships between the sequences is presented in Figure 2.

### 3.3. Genetic Diversity among the SARS-CoV-2 Variants

The mutations (substitutions) varied among the different individual clades represented in the data, resulting in distinct mutation means. The mutation means ranged from 17 mutations/genome and 11 mutations/all proteins to 100.7 mutations/genome and 68.9 mutations/all proteins, as compared to the reference genome. Notably, the Omicron subvariants exhibited the highest genetic diversity. These variants displayed a range of mutations, with XBB.1.16, XBB.2.3, XBB.1.9, XBB (22F), XBB.1.5, CH.1.1, and BA.2.75 showing high mutation means compared to other variants, ranging from 87.8 mutations/genome to 100.7 mutations/genome. Specifically, XBB.1.16 had the highest mean of 100.7 mutations/genome and 68.9 mutations/all proteins among the clades, with a range of mutations from 87 to 106 across all the samples.

**Figure 3:**
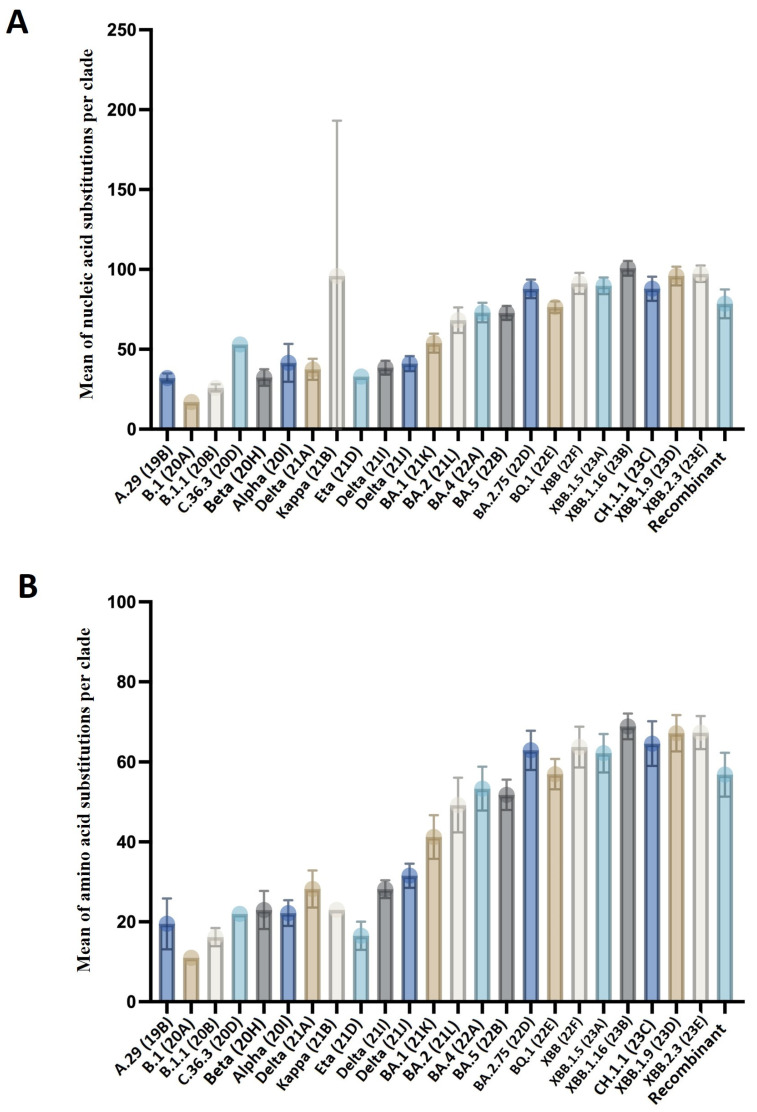
A boxplot represents the mean of mutations in the genomes and proteins of the different SARS-CoV-2 variants. Each dot represents the mean of mutations in viral genes or proteins. (**A**) represents the mean of nucleic acid mutations in the different clades, while (**B**) shows the mean of amino acid mutations in the different clades. The Omicron subvariants exhibited a higher mean of mutations than the other variants.

Most of the variants with a high mean of mutations were reported after November 2022. However, a surge in the mean of mutations occurred with the emergence of BA.1 at the beginning of December 2021. This variant had a mean of mutations of 53.6 mutations/genome, ranging from 42 to 98 mutations/genome across all the samples. In contrast, the variants observed before December 2021, including the Beta, Alpha, Kappa, Eta, and Delta clades, had a mean of mutations ranging from 30.7 to 41. Many other lineages with lower mutation means, such as A.29, B.1, and B.1.1, were reported at the beginning of April 2021, but disappeared before November 2021. The mutation mean per variant at the nucleic acid and amino acid levels is presented in Supplemental Table S1. The mean numbers of amino acid and nucleic acid substitutions in the various variants are shown in Figure 3.

### 3.4. Amino Acid Mutation Analysis across the Variants of SARS-CoV-2

Through an in-depth analysis of the SARS-CoV-2 sequences, it was determined that twelve proteins, including non-structural, structural, and accessory proteins, exhibited mutations at the amino acid level. Notably, the non-structural polyproteins ORF1a and ORF1b had 1366 amino acid mutations. Among the structural proteins, 933 amino acid mutations were identified, with 649 occurring in the S protein, 186 in the N protein, 55 in the M protein, and 43 in the E protein. Additionally, 330 amino acid mutations were found in the accessory proteins (ORF3a, ORF6, ORF7a, ORF7b, ORF8, and ORF9b).

Comparing the frequencies of amino acid mutations among the proteins, the S protein exhibited the highest mutation frequency, accounting for 47.6% of the total amino acid mutation frequency. Following the S protein, ORF1a, N protein, and ORF1b displayed relatively high mutation frequencies. On the other hand, proteins such as ORF3a, ORF8, ORF7a, ORF6, and ORF7b appeared to be more conserved, exhibiting the lowest frequency and percentage of amino acid mutations. Table 2 provides an illustration of the amino acid mutation frequencies and percentages for the various SARS-CoV-2 proteins.

When comparing the frequency of amino acid mutations in each protein, it was observed that the S, ORF1a, N, ORF1b, and ORF9b proteins exhibited higher frequencies of amino acid mutations compared to the other proteins. Among these proteins, BA.1 and XBB.1.9 demonstrated the highest frequencies of amino acid mutations. Notably, the S protein consistently had the highest frequency of amino acid mutations across all the detected variants. Most of the clades showed mutations in the ORF1a, ORF1b, S, and N proteins, with the exception of the B.1 clade, where no mutations were detected in the N protein. It is worth mentioning that all the proteins displayed higher mutation frequencies for the Omicron subvariants compared to the other variants. However, ORF7a, ORF7b, and ORF8 exhibited different patterns, with the highest mutation frequencies observed for Delta (21J).

Among the structural proteins (S, E, M, and N), the E and M proteins had lower frequencies of amino acid mutations compared to the S and N proteins. The highest frequency of amino acid mutations in the E and M proteins was observed for BA.1. Conversely, ORF7a, ORF6, and ORF7b had low frequencies of amino acid mutations across almost all the variants. Amino acid mutations in ORF7b were only detected in 12 out of 24 clades. Notably, no mutations were associated with the ORF10 protein across all the variants.

**Figure 4:**
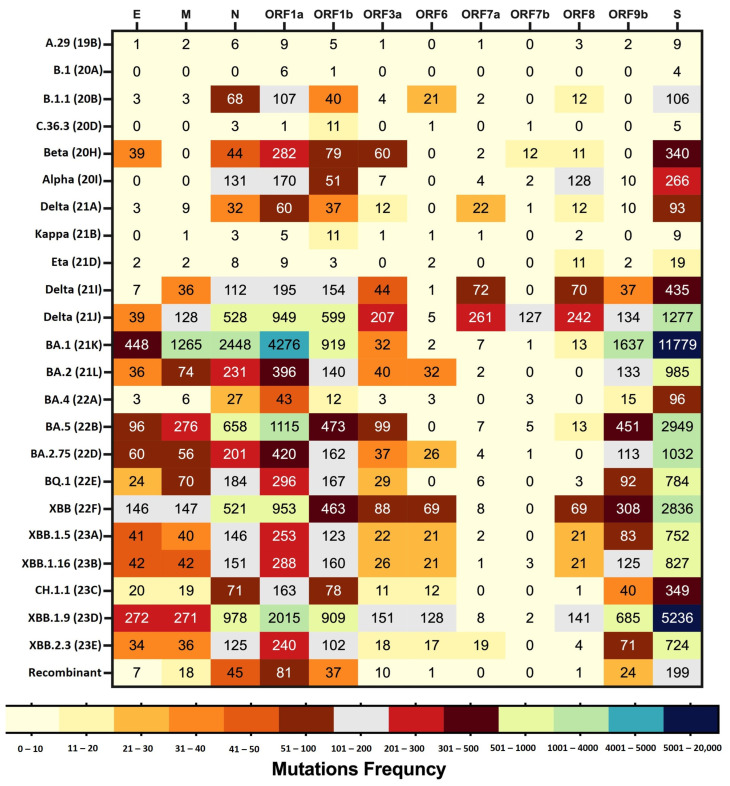
The heat map displays the frequency of amino acid mutations in each protein per SARS-CoV-2 clade. The different color codes present the differences in the frequency of mutations in each protein across the various SARS-CoV-2 clades.

Figure 4 presents the frequency of amino acid mutations in each protein per SARS-CoV-2 clade.

The analysis also detected various types of amino acid mutations, including substitutions, deletions, insertions, and stop codon mutations. Substitution mutations were the most prevalent type, observed in all the mutated proteins across all the variants. However, they were particularly frequent in the S protein. Among the substitution mutations in the dataset, the most commonly found were S:D614G (*n* = 1110), ORF1b:P314L (*n* = 1100), ORF1a:T3255I (*n* = 987), N:R203K (*n* = 894), S:P681H (*n* = 889), S:K417N (*n* = 887), N:G204R (*n* = 886), and S:H655Y (*n* = 881). In addition to substitutions, deletions were identified in all the proteins except ORF7b, with the highest prevalence observed in the S protein region. Deletions were present in all clades except A.29, B.1, and C.36.3. The most common deletion mutations were ORF1a:G3676- (*n* = 909), ORF1a:S3675- (*n* = 909), N:E31- (*n* = 860), N:R32- (*n* = 860), and N:S33- (*n* = 858). Insertion mutations were only observed in the S protein of the BA.1 clade, specifically involving the insertion of glutamic acid in two positions and proline in one position. Stop codon mutations were noted in three proteins, namely ORF8, ORF7b, and the M protein region. However, they were most frequently found in ORF8. These mutations were observed in seven clades, including Beta, Alpha, BA.1, XBB, XBB.1.5, XBB.1.16, and XBB.1.9. The most common stop codon mutations were ORF8:G8* (*n* = 237) and ORF8:Q27* (*n* = 28).

The analysis results for the frequencies of the different types of mutations in each protein for each clade, and the frequencies of the top 10 amino acid mutations in each protein are given in Supplemental Tables S2–S4.

#### 3.4.1. Amino Acid Mutations in Non-Structural Polyproteins

**Figure 5:**
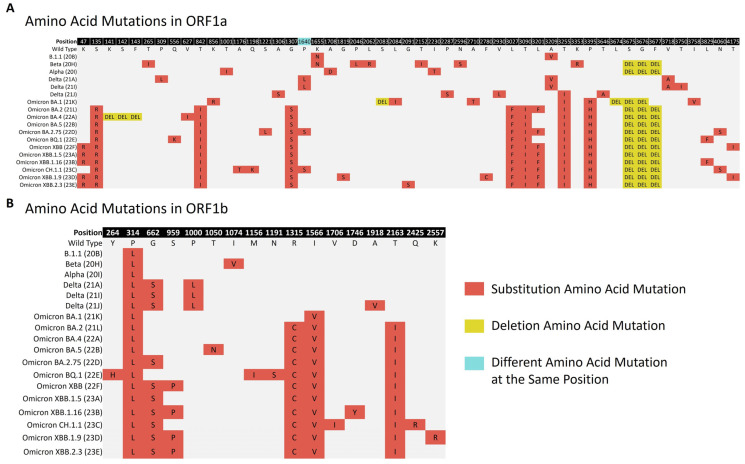
The major amino acid mutations frequently found in the non-structural polyproteins are depicted in the figure above. Panel (**A**) represents the ORF1a mutations, while panel (**B**) represents the ORF1b mutations across the various lineages from 1 April 2021 to 31 July 2023. Each type of mutation and the presence of different mutations at the same position are represented by a specific color.

The ORF1a polyprotein (4405 amino acids) and ORF1b polyprotein (2595 amino acids) were found to have substitution and deletion mutations. The most common substitution in ORF1a was ORF1a:T3255I (*n* = 987), which was found in Delta (21J) and all Omicron subvariants. A deletion at positions 3675–3677 was frequently observed in the study lineages, including the Alpha, Beta, and Omicron subvariants. However, in Omicron BA.1, the deletion started at positions 3674–3676. Notably, the sequence of ORF1a was similar in both Omicron BA.4 and Omicron BA.5, but Omicron BA.4 exhibited a unique deletion at positions 141–143. For ORF1b, the most frequently detected mutation was ORF1b:P314L (*n* = 1100), which was present in all the lineages. It was followed by ORF1b:I1566V (*n* = 860), which was frequently observed in the Omicron subvariants. A summary of the frequently detected amino acid changes in the ORF1a and ORF1b polyproteins is illustrated in Figure 5.

#### 3.4.2. Amino Acid Mutations Analysis of Structural Proteins (S, E, M, and N)

The amino acid mutations in the S, E, M and N proteins were investigated in the different lineages.

The S protein consists of 1273 amino acids. Amino acid changes were observed within 18–1264 regions of the protein. Different S protein mutation patterns were noticed among the study variants. The number of mutations in the protein was the lowest in the Nextstrain clade B.1.1, with only four amino acid changes frequently detected. However, the number of mutations was higher in the Omicron subvariants. For example, 41 amino acid changes were frequently observed in the S protein of Omicron XBB.2.3. In the case of substitutions within the variants, the most frequently found amino acid mutation in the protein was D614G (*n* = 1110). It was present in almost all of the variants. Some unique amino acid substitutions were observed with specific variants. S:F157S was exclusive to the B.1.1 clade; S:A570D, S:T716I, S:S982A, and S:D1118H to Alpha; S:D215G to Beta; S:R158G, S:A222V, and S:D950N to the Delta clades; S:Y145D, S:L212I, S:S371L, S:N856K, and S:L981F to Omicron BA.1; and S:V83A, S:H146Q, S:Q183E, S:V213E, S:G252V, S:L368I, and S:V445P were exclusive to the Omicron XBB variants. There were mutations not related to the Nextstrain or Pango lineages of the variant, but which were frequently observed with its sequences. S:L18F (*n* = 15) was repeatedly found in Beta; S:Q675K (*n* = 13) and S:H1101Y (*n* = 5) in Delta (21J); S:C1250F (*n* = 15) in Delta (21I); and S:R346K (*n* = 262) and S:F643L (*n* = 16) in Omicron BA.1. Different amino acid changes at the same position were observed among the different variants. These positions included 19,142, 157, 213, 339, 346, 371, 478, 484, 486, 501, and 681 of the protein. Notably, an amino acid change at position 501 showed up in all the variants, except the Delta clades. The asparagine (N) amino acid in this position converted into threonine (T) for B.1.1, while it converted into tyrosine (Y) for the Alpha, Beta, and Omicron subvariants. In the instance of deletion mutations in the S protein, deletions in S:L24-, S:P25-, and S:P26- were found in all the Omicron subvariants except Omicron BA.1. Deletion mutations unique to Omicron BA.1 were detected, including S:G142-, S:V143-, and S:N211-. Unique deletions in the Delta and Beta variants were also observed, including S:E156- and S:F157- in the Delta clades and S:L241-, S:L242-, and S:A243- in Beta. The insertion of EPE amino acids was observed only with Omicron BA.1, and it was the only insertion mutation in our data.

For the remaining structural proteins, the E protein has 75 residues of amino acids. The protein showed changes within the 9–71 residues. The amino acid mutations in this protein were observed in the Beta and Omicron subvariants. The most common mutation in this protein was E:T9I (*n* = 866), which was observed in all the Omicron subvariants, followed by amino acid change E:T11A (*n* = 306).

The M protein, on the other hand, has 222 amino acids. Mutations were frequently observed in regions 3–104. All the lineages had an amino acid mutation in this protein, except for B.1.1 and Beta. The most common mutations were M:Q19E (*n* = 840) and M:A63T (*n* = 839), found in all the Omicron subvariants. At position 3 of the protein, different amino acid mutations were associated with the various variants. In this position, aspartic acid (D) was converted into glycine (G) in Omicron BA.1, and into asparagine amino acid (N) in Omicron BA.5 and Omicron BQ.1.

**Figure 6:**
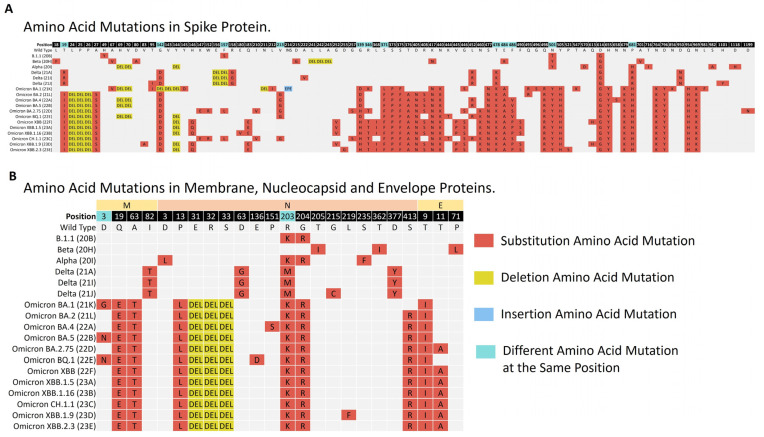
The major amino acid mutations that were frequently found in the structural proteins are illustrated. Panel (**A**) represents the spike (S) protein mutations, while panel (**B**) represents mutations in the membrane (M), nucleocapsid (N), and envelope (E) proteins across the various clades from 1 April 2021 to 31 July 2023. Each type of mutation and the presence of different mutations at the same position are represented by a specific color.

Finally, the N protein comprises 419 amino acids. The mutations were detected within the 3–413 regions. The most common amino acid mutations in the protein were N:R203K (*n* = 894), followed by N:G204R (*n* = 886), N:P13L (*n* = 863), N:E31-, N:R32-, and N:S33- (*n* = 860–858). All these mutations were found in all the Omicron subvariants. The two consecutive mutations, N:R203K and N:G204R, were also found in B.1.1 and Alpha. A set of mutations unique to the Delta clades were observed frequently, including N:D63G, N:R203M, N:D377Y, and N:G215C. A summary of the frequently detected amino acid changes in the S E, M, and N proteins is illustrated in Figure 6.

##### Amino Acid Mutations Analysis of the Regions of the Structural Proteins

The most variable region among the SARS-CoV-2 structural proteins regions was investigated. Among the regions of structural proteins, the S protein regions frequently had mutations, with the N-terminal S1 subunit being the most likely to have mutations. The most common mutations in the N-terminal S1 subunit were S:K417N, S:D614G, S:H655Y, and S:P681H. This region was followed by the C-terminal S2 subunit of the S protein, which often consisted of S:N764K, S:D796Y, S:Q954H, and S:N969K mutations. Conversely, the E protein and M proteins were more conserved than the other structural proteins. The N-terminal domain of the E protein had the lowest number of mutations, with no mutations found in this region.

The frequency of amino acid mutations within the regions of a single protein was compared. In the N protein, amino acid mutations were most frequently detected in the N-arm region, with N:P13L being the most often found amino acid mutation. This region was followed by the SR-rich region, with N:R203K and N:G204R being the most observed mutations in the region. In contrast, the C-terminal domain region was more conserved, and the frequency of amino acid mutations was the lowest in this region of the protein. Among the regions of the M protein, the N-terminal ectodomain region most frequently had amino acid mutations, with M:Q19E being the mutation most often noticed. The N-terminal ectodomain region was followed by the triple-membrane-spanning domain region. The M:A63T mutation was the most frequently detected in this region. For the E protein, the transmembrane domain had the highest number of mutations. The E:T9I mutation was the most prevalent in this region. In contrast, the remaining regions of the protein were more conserved. The frequency of amino acid mutations in the domains of each structural protein during the study period is shown in Table 3.

#### 3.4.3. Amino Acid Mutations Analysis of Accessory Proteins

**Figure 7:**
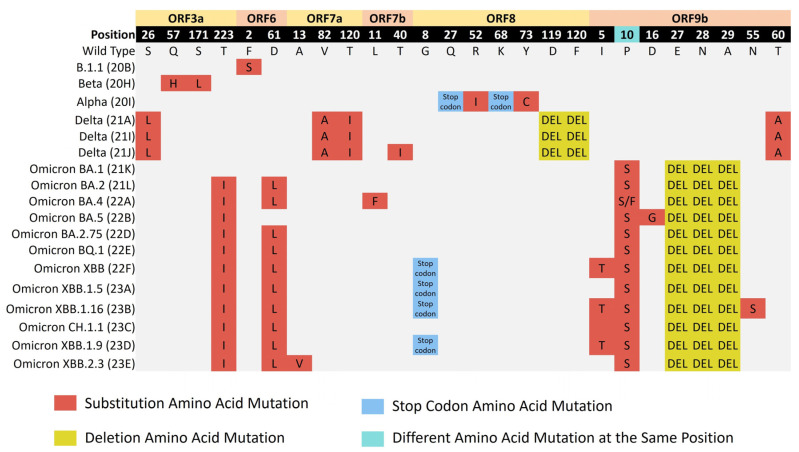
The major amino acid mutations that were frequently found in the accessory proteins across the clades from 1 April 2021 to 31 July 2023. Each type of mutation and the presence of different mutations at the same position are represented by a specific color.

Substitution, deletion, and stop codon amino acid mutations were reported in the accessory proteins. The presence of mutations in some accessory proteins was associated with specific variants. Mutations in ORF3a were found in the Beta and Delta clades and the Omicron subvariants, except Omicron BA.1. The most common mutation in the protein was ORF3a:T223I (*n* = 451). It was detected in all the Omicron subvariants except Omicron BA.1. Amino acid changes in ORF6 were identified in B.1.1 and the Omicron subvariants, except Omicron BA.1 and Omicron BA.5. Two mutations were frequently detected in the protein, including ORF6:D61L (*n* = 326) and F2S (*n* = 23). For ORF7a, the mutations in this protein were frequently found in the Delta clades, where they showed the substitutions ORF7a:T120I (*n* = 170) and ORF7a:V82A (*n* = 165), which were the most detected. For ORF7b, the substitution ORF7b:T40I (*n* = 126) was the most frequently detected mutation in the protein and was found with Delta (21J). The ORF8 protein had substitutions, deletions, and stop codon amino acid mutations. The protein frequently carried mutations in the Alpha and Delta clades and the Omicron XBB variants, except XBB.2.3. The stop codon mutation ORF8:G8* (*n* = 237), which was associated with the Omicron XBB variants, was the most detected in the protein. This mutation was followed by the deletion mutations ORF8:D119- (*n* = 154) and ORF8:F120- (*n* = 154), which were observed in the Delta clades. The protein had unique stop codon mutations associated with Alpha, including Q27* (*n* = 28) and K68* (*n* = 22). Amino acid mutations of the ORF9b protein were frequently detected in the Delta and Omicron variants. The deletions ORF9b:E27-, ORF9b:N28-, and ORF9b:A29-, along with P10S, were the most common mutations in the protein. These mutations were found in all Omicron subvariants. The frequently detected amino acid changes in the SARS-CoV-2 accessory proteins are illustrated in Figure 7.

## 4. Discussion

This genomic surveillance and mutation analysis study investigated the genome sequences of SARS-CoV-2 from COVID-19 patients in Saudi Arabia from 1 April 2021 to 31 July 2023. Variations in clinical and demographic patterns have been noted among countries. The mean age of COVID-19 patients differs in different geographic regions [26]. Nonetheless, data from several countries have shown that elderly patients (≥60 years old) have a higher risk of severe outcomes, including hospitalization, ICU admission, and death rates, compared to young and middle-aged patients [27,28]. Furthermore, a study from the United States revealed an increase in the percentage of hospitalizations, ICU admissions, and fatalities among older persons (65 and older), with a majority of COVID-19 deaths occurring among them. These data emphasize the role of age as a risk factor for severe COVID-19 outcomes [29]. During the research period, 11.7% of the patients had a severe illness. A total of 4.1% of those patients died. According to several studies, the severity of COVID-19 has altered over time. They have indicated that the severity of the disease has changed due to reasons such as the emergence of different SARS-CoV-2 variants and the accessibility of vaccinations. For example, studies conducted in the United States, France, California, and the Kingdom of Belgium included data on COVID-19 patients during the Alpha, Delta, and Omicron circulating periods. The studies showed a reduced risk of hospitalization, ICU admission, and death in patients with an infection of the Omicron variant compared to the other variants [29,30,31,32,33]. Another study noted the impact of SARS-CoV-2 variants on the severity of COVID-19. There was a decline in the severity of the disease from the pre-Alpha period to Omicron’s predominance [34]. Finally, a study on COVID-19 pediatric patients found that the disease’s severity decreased over the course of the SARS-CoV-2 pandemic [35].

The genomic surveillance showed that the distribution of the SARS-CoV-2 variants circulating in Saudi Arabia was similar to that of global statistics reports from similar periods. Alpha was the most dominant variant in a similar interval of our study (April–May 2021) in several regions, such as Finland, the United Kingdom, the United States, and Denmark [36,37,38,39]. Variant distribution reports showed a decrease in Alpha and Beta variant infections beginning in June, as well as an increase in Delta cases [40,41]. The reports on COVID-19 infections from July to November 2021 showed increased frequencies of Delta variant infections worldwide [40,42,43]. Notably, an investigation of South American sequences revealed that in Brazil, the Delta variant displaced the Gamma variant, while in Colombia, the Delta variant replaced the Mu variant [43]. In our findings, Omicron BA.1 was the most common circulating variant beginning in December 2021. This agrees with several SARS-CoV-2 transmission rate reports from several countries, such as the Netherlands, Turkey, Germany, and Norway [44,45,46,47]. Omicron BA.4 and Omicron BA.5 were found for the first time in South Africa in January 2022 [48]. They comprised more than 50% of the sequenced cases in South Africa and were responsible for the fifth wave of COVID-19 in the country [49]. The proportion of cases in the United States with Omicron BA.4 and BA.5 infections increased in March 2022, and after that, the two lineages dominated the nation [50]. Regarding Omicron XBB, the first observation of the variant was in India in August 2022. It is a recombinant of Omicron BA.2, BJ.1, and BA.2.75 [51,52]. The variants circulated worldwide in January 2023. However, in South and Southeast Asian nations like India, Singapore, and Indonesia, Omicron XBB was the most common form [52]. Overall, our data show that the virus entered into Saudi Arabia numerous times, and the patterns of the SARS-CoV-2 variants show how the virus has evolved over time, with different variants identified at various times of the COVID-19 pandemic. Furthermore, the Delta and Omicron variants are more highly transmissible than the other variants, with Omicron being more transmissible than Delta, according to our results from Saudi Arabia and data from earlier publications from multiple countries.

In this study, the mutation mean of each variant was compared. The highest mean of mutation was observed in the Omicron subvariants. This finding is in line with research that analyzed SARS-CoV-2 variants from isolates in India and found that the rate of mutation increased significantly from the Alpha to Omicron variants [53]. The Omicron variants were found to contain more mutations than the other variants, with 50 mutations detected throughout the genome. In addition, the S protein contained at least 32 mutations. This number is twice the number of S protein mutations detected in the Delta variant [54]. Because of the high mutation load carried by the Omicron variants, researchers have found that their infectivity and immune escape are comparatively higher than those of the other variants [55].

The proteins of SARS-CoV-2 with a high frequency of amino acid mutations were determined to understand the pattern of SARS-CoV-2 evolution. The ORF1a and ORF1b proteins are associated with high mutation frequencies. There are differences between the observations of the frequency of mutations in ORF1a and ORF1b presented here and those from other data [18,56]. The findings of one research study, which examined the mutations in SARS-CoV-2 genes and proteins present in Indian isolates, identified ORF1a and ORF1b as the proteins with a high mutation rate [56]. However, another study contradicted these findings, suggesting that ORF1a and ORF1b exhibited low levels of mutations [18]. The disparities in these findings could be attributed to differences in the data analysis methods, variations in the genome sequences used in reports collected at different times, discrepancies in the data collection of local or global isolates, or the focus of certain reports solely on specific variants that were more clinically relevant. Furthermore, when SARS-CoV-2 spreads, the mutation profile of the genes or proteins changes, resulting in differences in the findings among publications [18,56]. ORF1a:T3255I was the most detected mutation in ORF1a, while ORF1b:P314L and ORF1b:I1566V were the most common mutations in ORF1b. The mutations in ORF1a and ORF1b have roles in viral replication. As a result, viral pathogenicity, the capacity to evade host immunity, and the development of resistance to commonly used antiviral treatments may all be affected [57,58].

The highest frequency of mutations was detected in the S protein across all the studied variants. The S protein is in charge of both viral attachment and binding to the angiotensin-converting enzyme 2 (ACE2) receptor in a host cell, as well as the membrane fusion process essential for cell entry [59]. Thus, SARS-CoV-2 variants with multiple spike mutations enabled increased transmission and impacted the neutralizing activity of antibodies [60]. S:D614G, which was the most frequently detected mutation in the protein, was reported to promote a conformational change in the S protein, causing it to become more open and provide a greater area of contact with the ACE2 receptor, possibly improving its protein binding affinity and interaction with the ACE2 receptor [61]. The increasing fitness, infectivity, and transmissibility of the virus due to this mutation were also reported [62]. There is evidence that S:H655Y, S:P681H, and S:K417N confer resistance or allow for the escape of variants from immune responses, thus enhancing viral replication and transmission [63,64]. The association between mutations in the S protein of SARS-CoV-2 and patient characteristics was investigated in a previous work. Certain mutations, including H69-, K417N, N440K, S477N, and N501Y, were associated with younger, female patients, while L452R and P681R were found in older patients. Additionally, patients with H69-, K417N, and D614G had the highest viral load, and those with E484K and N501Y had the lowest. Furthermore, the association between mutations in the S protein and vaccination status was studied. N440K was mostly detected in unvaccinated patients, whereas L452R and P681R were mostly found in vaccinated patients [65].

The N-terminal S1 subunit of the S protein was the most variable region among the regions of the structural proteins. In the S1 subunit, the receptor-binding domain (RBD) is a region ranging from 319 to 541 residues that binds and interacts with the ACE2 receptor on the host cell to start the viral entry process [55,64,66,67,68]. Recent research has discussed the key residues involved in the interaction between the RBD and ACE2. One group found 13 essential interacting residues in the RBD, including Q493, Y505, Q498, N501, T500, N487, Y449, F486, K417, Y489, F456, Y495, and L455 [66]. We frequently identified twelve amino acid mutations in eight positions of these amino acid residues, including S:K417T, S:K417N, S:Y449H, S:F456L, S:F486V, S:F486S, S:F486P, S:Q493R, S:Q498R, S:Y505H, S:N501Y, and S:N501T. Some of these mutations were studied to investigate their potential effect on the binding affinity of the RBD to ACE2. The RBD amino acid mutations that were observed in our study and reported to affect its binding affinity with ACE2 include N501T, N501Y, G339D, N440K, S477N, T478K, Q493K, G446V, and E484K, and were among the mutations that enhanced the binding affinity, while S371L, S373P, S375F, K417N, G446S, E484A, G496S, Q498R, and Y505H were among the mutations that reduced the binding affinity. Notably, N501Y has been noted in studies to be associated with a high binding affinity between the RBD and ACE2 in all SARS-CoV-2 variants, whereas K417N possessed the lowest binding affinity between the ACE2 and RBD [66,67,68,69]. As these mutations have the potential to affect protein function, they might alter viral characteristics, such as infectivity and transmissibility.

Another region in the N-terminal S1 subunit of the S protein, called the N-terminal domain (NTD), possessed the most indel mutations, such as S:L24-, S:P25-, S:P26-, S:E156-, S:F157-, S:H69-, S:V70-, G142-, S:N211-, S:L241-, S:L242-, S:A243-, and the insertion of EPE at position 214. S:H69- and S:V70- mutations appeared in the Alpha variant; after that, it reappeared with some of the Omicron subvariants, including BA.1, BA.4, BA.5, BQ.1. S:H69-, and S:V70-, which were reported to be associated with failure detection or a dropout of the S gene target in the TaqPath test for quantitative reverse transcription PCR (RT-qPCR) detection, with no effect on the other two gene targets: ORF1ab and N [70]. The insertion of EPE at position 214 was reported to be associated with induced structural changes in the region and antibody evasion [71]. The N-terminal domain (NTD) of the S1 subunit is known as a point of recognition for vaccines and the immune response. Therefore, several mutations in this region had effects on viral immunity, leading to an evasion of immunity and a decreased effect of vaccine-induced immunity [53,72].

The C-terminal S2 subunit of the S protein was more conserved. It is thought to be highly conserved among coronaviruses. As a result, the S2 subunit could be promising for the development of vaccines [19]. The S2 subunit held relatively fewer mutations compared to the S1 subunit in most of the SARS-CoV-2 variants. Yet, Omicron had many more mutations in S2 in comparison to the other variants. Mutations in the S2 subunit may impact fusion activity and virulence. S:Q954H and S:N969K were reported to reduce fusion effectiveness. S:N764K and S:D796Y seem to be involved in affecting spike protein cleavability and could impact protein stability [72].

For the structural proteins (N, E, and M), the N protein has a variety of essential roles in the infectious cell, such as RNA genomic packing, viral transcription, and assembly [73]. The N-arm of the N protein is responsible for RNA binding and packaging the viral genome into a helical ribonucleocapsid (RNP) during viral self-assembly. The SR-rich region plays a role in regulating the activity and localization of the nucleocapsid protein [74,75]. Based on our previous study, the mutations observed in the N-arm and SR-rich regions were found to be linked with decreased disease severity. Specifically, the mutations in the SR-rich region were associated with a lower risk of mortality, while the mutations in the N-arm region were associated with a reduced likelihood of ICU admission [76]. The two consecutive amino acid substitutions (N:R203K and N:G204R) are located in the SR-rich region. They are associated with viral virulence and pathogenesis. They were reported to be associated with an increased viral load and severity of disease in the data of patients from Saudi Arabia during the early wave of COVID-19 [77]. In addition, they were associated with an enhanced inflammatory immune response in severe cases of COVID-19 [78]. Finally, there was the potential impact of these mutations on the ability of the SARS-CoV-2 nucleocapsid protein to transfer to neighboring non-infected cell surfaces [79].

Among the regions of the E protein, the transmembrane domain had the highest frequency of mutations. However, two studies have revealed that the most frequent mutations were detected in the C-terminal domain (CTD) of the E protein [80,81]. The differences between the observations of these studies with our results could be due to the time at which the two studies were conducted, which was before the appearance of the Omicron variant. The E protein functions as a viroporin, which creates an ion channel in the cell membranes. Ion channels may operate as virulence factors, playing a part in the viral life cycle and various processes, such as pathogenesis [82,83]. Therefore, acquiring mutations in the protein might have important implications for the structure and ion channel activity of the virus. Some mutations in the protein have been reported to affect the virulence and pathogenicity of SARS-CoV-2. A study that analyzed the mutations in the E viroporin of SARS-CoV-2 from deceased and surviving COVID-19 patients found a correlation between the mutations in the viroporin and patient mortality. In addition, the study mentioned that T9I, F4L, P71L, V49L, P71S, S68F, and R69I were associated with disease severity and mortality [82]. Among these mutations, E:T9I and E:P71L were frequently detected in our data.

In the case of the M protein, the protein is involved in multiple viral functions, such as the initial attachment to the host cell and viral protein assembly, in conjunction with the N and E proteins [84]. Therefore, mutations in this protein could affect viral assembly and pathogenicity. M:Q19E and M:A63T were among the first five mutations with the highest frequency in the M protein from a study that included a global analysis to investigate the frequencies of mutations in the structural proteins of SARS-CoV-2 from different continents. It has been reported that the two mutations impacted the stabilization of the M protein [22,85]. According to our study, the E and M proteins are more conserved than the S and N proteins. This was mentioned in another research study, as the two proteins are highly conserved structural proteins of SARS-CoV-2, and have been recommended as prospective targets for developing cross-protective vaccines [86].

Amino acid mutations were found in the following accessory proteins of SARS-CoV-2 sequences with varying frequencies: ORF3a, ORF6, ORF7a, ORF7b, ORF8, and ORF9b. ORF9b was the accessory protein with the highest number of mutation frequencies among the accessory proteins. ORF9b is found on the mitochondrial membrane and interacts with the receptor protein TOM70, a surface receptor for the gateway of protein imports into the mitochondria, called the translocase of the outer membrane (TOM) complex. ORF9b reduces the immune response of infected cells by targeting TOM70 and multiple molecules of innate antiviral signaling pathways. As a result, it improves immune response evasion and facilitates viral replication [87,88,89,90,91]. Mutations in this protein have been associated with high hospitalization and mortality rates [92]. ORF9b:E27-, ORF9b:N28-, ORF9b:A29-, and ORF9b:P10S may impact protein stability and the interaction with other signaling pathways proteins [93]. In addition, ORF9b:P10S has been included among the amino acid mutations significantly associated with increased viral fitness [94].

Among the SARS-CoV-2 accessory proteins, ORF3a is the largest, with 275 amino acid residues in length. The protein can induce necrotic cell death and lysosomal damage. Thus, it has the capability to cause autophagy or the apoptosis of infected cells [88]. ORF3a has possible effects on immune evasion pathways. The protein enhances the release of cytokine and cytokine storm. And this enhancement is possibly mediated by ORF3a:T223I in ORF3a of Omicron [93]. ORF3a:T223I is predicted to be protein destabilizing [95]. Investigating whether there is an association between this mutation and viral attenuation, transmission, or virulence is necessary, as high transmissibility was associated with Omicron BA.1, the variant that lacks this mutation [96]. Through a mutation analysis of ORF8, the most frequently detected mutations in the protein were ORF8:G8*, ORF8:D119-, and ORF8:F120-. The deletions ORF8:D119- and ORF8:F120- have been predicted to result in the structural instability of the ORF8 dimer [97]. Stop codon mutations in the protein were frequently associated with five clades. Alpha had Q27* and K68*, while XBB (22F), XBB.1.9, XBB.1.5, and XBB.1.16 possessed G8*. The defective ORF8 in the viral strains has been identified in immunocompromised patients who were chronically infected with SARS-CoV-2. These strains do not effectively suppress the immune response and cause a milder disease but longer infection, with a higher potential for transmission [98].

Regarding the viral proteins that could potentially serve as targets for developing vaccine-induced immune responses and therapeutics, several viral proteins might act as potential targets, such as non-structural proteins S, N, and ORF3a [99,100,101]. The S protein has been the main target of the majority of vaccines, such as recombinant adenovirus vectors and mRNA (Pfizer and Moderna) vaccines. The purpose of these vaccines is to induce neutralizing antibodies against the S protein [100,102]. According to our observations, the S protein had the highest mutation frequency. This has been noted in other studies, in which some variants have been found to hold a heavily mutated S protein, which allows them to evade vaccine-induced antibodies with a high efficiency [54,103]. The S protein has a highly mutated nature, which was revealed through the high number of mutations that were associated with the emergence of the Omicron variants. Thus, the neutralizing activity of the antibodies induced by vaccines against SARS-CoV-2 variants was reduced [54]. According to our data, in addition to the S protein, the ORF1a, N, and ORF1b proteins of SARS-CoV-2 had a mutation frequency higher than those of other proteins across all the study variants. So, due to their high mutations, the possibility of them being perfect targets for developing vaccines or therapeutics may decrease [18]. In contrast, the ORF3a, ORF8, ORF7a, ORF6, and ORF7b viral proteins were associated with a lower mutation frequency than other proteins, and were relatively conserved proteins. Considering the increased conservancy of these proteins, they may be a promising immunogenic approach for the development of a vaccine [19].

There are limitations to this study. In this study, the mean, percentage, and frequency of mutations were compared at the levels of whole genes and proteins in different SARS-CoV-2 variants. However, the variants found during late pandemic times are predicted to contain more substitutions than those collected during the early pandemic periods. In addition, the decreased sample size of some variants could affect the mutation frequency results of the variant’s gene or protein. Therefore, the data only show a single facet of the evolution of SARS-CoV-2. Considering the various sampling dates of the sequences, evolutionary analyses and methods must be integrated to fully comprehend the variance in the substitution rate among lineages.

## 5. Conclusions

This study provides valuable insights into the whole genome of SARS-CoV-2 variants. Our findings emphasize that the virus is continuously evolving and acquiring mutations in its genome. These mutations have the potential to affect various aspects of the virus, including its transmission, pathogenesis, and virulence. Therefore, it is crucial to conduct further research to understand the evolution of SARS-CoV-2 and the incidence of mutations in its viral proteins.

By gaining a deeper understanding of these mutations, scientists can identify potential targets for improving molecular diagnostic tests, enhancing sequencing and mutation analysis methods, and developing effective therapeutic interventions and vaccines against SARS-CoV-2 and future coronaviruses. However, it is important to consider several factors when selecting a protein or gene as a target for vaccine or drug development against SARS-CoV-2. Further investigations, including large-scale analyses of mutations in SARS-CoV-2 genes or proteins, coupled with clinical trials, are necessary to validate the effectiveness and suitability of the chosen targets for specific applications. This comprehensive approach will contribute to the development of more robust strategies with which to combat SARS-CoV-2 and future coronaviruses effectively.

## Figures and Tables

**Table 1:** Variants detected in our cohort.

Clade	*N* (%)
A.29 (19B)	3 (0.3)
B.1 (20A)	1 (0.1)
B.1.1 (20B)	19 (1.6)
C.36.3 (20D)	1 (0.1)
Beta (20H)	24 (2.1)
Alpha (20I)	24 (2.1)
Delta (21A)	12 (1.0)
Kappa (21B)	1 (0.1)
Eta (21D)	2 (0.2)
Delta (21I)	34 (2.9)
Delta (21J)	139 (12.0)
Omicron BA.1 (21K)	430 (37.0)
Omicron BA.2 (21L)	33 (2.8)
Omicron BA.4 (22A)	4 (0.3)
Omicron BA.5 (22B)	94 (8.1)
Omicron BA.2.75 (22D)	34 (2.9)
Omicron BQ.1 (22E)	23 (2.0)
Omicron XBB (22F)	80 (6.9)
Omicron XBB.1.5 (23A)	18 (1.6)
Omicron XBB.1.16 (23B)	21 (1.8)
Omicron CH.1.1 (23C)	11 (1.0)
Omicron XBB.1.9 (23D)	133 (11.5)
Omicron XBB.2.3 (23E)	16 (1.4)
Recombinant	4 (0.3)

**Table 2:** Frequencies of detected amino acid mutations in each protein.

Protein	*N* (%)
S	31,182 (47.6)
ORF1a	12,369 (18.9)
N	6739 (10.3)
ORF1b	4751 (7.2)
ORF9b	3977 (6.1)
M	2503 (3.8)
E	1327 (2.0)
ORF3a	905 (1.4)
ORF8	784 (1.2)
ORF7a	429 (0.6)
ORF6	366 (0.5)
ORF7b	158 (0.2)
Total	65,490

**Table 3:** The frequency of amino acid mutations in the domains of the SARS-CoV-2 structural proteins.

Protein	Functional Domain	Range	*N* (%)
Spike	Signal peptide	Residues 1–13	4 (0.01)
	N-terminal S1 subunit	Residues 14–685	26,431 (63.3)
	C-terminal S2 subunit	Residues 686–1273	4757 (11.4)
Nucleocapsid	N-arm	Residues 1–43	3497 (8.4)
	NTD (RNA binding domain)	Residues 44–174	322 (0.8)
	SR-rich region	Residues 175–204	1966 (4.7)
	LKR	Residues 205–254	224 (0.5)
	CTD (dimerization domain)	Residues 255–364	56 (0.1)
	C-tail	Residues 365–419	679 (1.6)
Membrane	N-terminal ectodomain	Residues 1–25	1366 (3.3)
	Triple-membrane-spanning domain	Residues 26–101	1127 (2.7)
	C-terminal endodomain	Residues 102–222	11 (0.03)
Envelope	N-terminal domain	Residues 1–8	0
	Transmembrane domain	Residues 9–38	1272 (3.1)
	C-terminal tail	Residues 39–75	55 (0.1)

## Data Availability

The data and codes presented in this study are available on request from the corresponding author. The data are not publicly available due to privacy restrictions. The SARS-CoV-2 sequences were deposited into the GISAID website.

## Supplementary Materials

The following supporting information can be downloaded at: https://www.mdpi.com/article/10.3390/microorganisms12030467/s1, Table S1: mutations mean per clade/variant at the nucleic acid and the amino acid levels; Table S2: frequencies of different type of mutations found in SARS-CoV-2 proteins; Table S3: frequencies of different type of mutations in each protein per each clade; Table S4: frequencies of the top 10 amino acid mutations in each protein.
