# Supplementary material for: Genomic Surveillance and Mutation Analysis of SARS-CoV-2 Variants among Patients in Saudi Arabia
Source: Microorganisms. 2024 Feb 26;12(3):467. doi: 10.3390/microorganisms12030467 (PMC10972307; doi:10.3390/microorganisms12030467)
Supplement: Supplementary file 1 [file microorganisms-12-00467-s001.zip › microorganisms-2855107-supplementary.pdf]

Table S1. Mutations mean per clade/variant at the nucleic acid and the amino acid levels.

Clades	Mean of nucleic acid mutations (substitutions).	Minimum	Maximum	Mean of amino acid mutations (substitutions).	Minimum	Maximum	Number of Samples
A.29 (19B)	32	30	34	19.5	15	24	2
B.1 (20A)	17	17	17	11	11	11	1
B.1.1 (20B)	25.8	21	29	16.2	11	19	22
C.36.3 (20D)	53	53	53	22	22	22	1
Beta (20H)	30.7	25	40	21.2	15	27	23
Alpha (20I)	38.5	32	53	22.1	18	29	21
Delta (21A)	37.4	30	54	28.2	23	39	9
Kappa (21B)	33	33	33	23	23	23	1
Eta (21D)	36	32	40	16.5	14	19	2
Delta (21I)	37.8	32	48	27.7	24	32	32
Delta (21J)	41	29	57	31.5	19	40	120
BA.1 (21K)	53.6	42	98	41	16	53	385
BA.2 (21L)	68.7	50	93	49.6	32	69	31
BA.4 (22A)	73	66	77	53.3	47	57	3
BA.5 (22B)	73.3	63	84	52.2	43	60	85
BA.2.75 (22D)	87.8	75	98	62.9	51	73	28
BQ.1 (22E)	76.4	67	84	57	48	61	23
XBB (22F)	90.9	68	105	63.5	44	73	71
XBB.1.5 (23A)	89.3	81	99	61.8	55	71	18
XBB.1.16 (23B)	100.7	87	106	68.9	60	73	21
CH.1.1 (23C)	87.9	78	97	64.6	57	72	10
XBB.1.9 (23D)	95.8	78	108	67.1	53	76	134
XBB.2.3 (23E)	96.9	89	108	67.3	61	77	15
Recombinant	86	82	91	61	58	65	3

Table S2. Frequencies of different type of mutations found in SARS-CoV-2 proteins.

Type of mutation	E	M	N	ORF1a	ORF1b	ORF3a	ORF6	ORF7a	ORF7b	ORF8	ORF9b	S	Grand Total
Deletion	66	67	2650	3353	64	8	2	17	0	349	2609	3982	13167
Insertion	0	0	0	0	0	0	0	0	0	0	0	383	383
stop codon	0	4	0	0	0	0	0	0	12	284	0	0	300
Substitution	1257	2430	4071	8979	4672	894	361	412	146	145	1363	26746	51477
Grand Total	1323	2501	6721	12332	4736	902	363	429	158	778	3972	31111	65327

Table S3. Frequencies of different type of mutations in each protein per each clade.

Variant/ Type of Mutation	E	M	N	ORF1a	ORF1b	ORF3a	ORF6	ORF7a	ORF7b	ORF8	ORF9b	S	Grand Total
A.29 (19B)	1	2	6	9	5	1	0	1	0	3	2	9	39
Substitution	1	2	6	9	5	1	0	1	0	3	2	9	39
B.1 (20A)	0	0	0	6	1	0	0	0	0	0	0	4	11
Substitution	0	0	0	6	1	0	0	0	0	0	0	4	11
B.1.1 (20B)	3	3	68	107	40	4	21	2	0	12	0	106	366
Deletion	0	0	0	4	10	0	0	0	0	0	0	14	28
Substitution	3	3	68	103	30	4	21	2	0	12	0	92	338
C.36.3 (20D)	0	0	3	1	11	0	1	0	1	0	0	5	22
Substitution	0	0	3	1	11	0	1	0	1	0	0	5	22
Beta (20H)	39	0	44	282	79	60	0	2	12	11	0	340	869
Deletion	12	0	0	93	26	0	0	0	0	2	0	87	220
stop codon	0	0	0	0	0	0	0	0	12	1	0	0	13
Substitution	27	0	44	189	53	60	0	2	0	8	0	253	636
Alpha (20I)	0	0	131	170	51	7	0	4	2	128	10	266	769
Deletion	0	0	35	70	0	0	0	0	0	23	10	75	213
stop codon	0	0	0	0	0	0	0	0	0	45	0	0	45
Substitution	0	0	96	100	51	7	0	4	2	60	0	191	511
Delta (21A)	3	9	32	60	37	12	0	22	1	12	10	93	291
Deletion	2	0	0	4	2	0	0	0	0	12	0	18	38
Substitution	1	9	32	56	35	12	0	22	1	0	10	75	253
Kappa (21B)	0	1	3	5	11	1	1	1	0	2	0	9	34
Deletion	0	0	0	5	6	0	0	0	0	0	0	0	11
Substitution	0	1	3	0	5	1	1	1	0	2	0	9	23
Eta (21D)	2	2	8	9	3	0	2	0	0	11	2	19	58
Deletion	0	0	2	6	0	0	2	0	0	9	0	6	25
Substitution	2	2	6	3	3	0	0	0	0	2	2	13	33
Delta (21I)	7	36	112	195	154	44	1	72	0	70	37	435	1163
Deletion	6	0	0	5	8	0	0	0	0	68	0	65	152
Substitution	1	36	112	190	146	44	1	72	0	2	37	370	1011
Delta (21J)	39	128	528	949	599	207	5	261	127	242	134	1277	4496
Deletion	22	0	2	4	5	0	0	8	0	230	0	225	496
Substitution	17	128	526	945	594	207	5	253	127	12	134	1052	4000
BA.1 (21K)	448	1265	2448	4276	919	32	2	7	1	13	1637	11779	22827
Deletion	23	56	1224	1602	3	2	0	0	0	1	1223	1577	5711
Insertion	0	0	0	0	0	0	0	0	0	0	0	383	383
stop codon	0	4	0	0	0	0	0	0	0	2	0	0	6
Substitution	425	1205	1224	2674	916	30	2	7	1	10	414	9819	16727
BA.2 (21L)	36	74	231	396	140	40	32	2	0	0	133	985	2069
Deletion	1	9	96	96	0	0	0	0	0	0	96	147	445
Substitution	35	65	135	300	140	40	32	2	0	0	37	838	1624
BA.4 (22A)	3	6	27	43	12	3	3	0	3	0	15	96	211
Deletion	0	0	9	18	0	0	0	0	0	0	9	15	51
Substitution	3	6	18	25	12	3	3	0	3	0	6	81	160
BA.5 (22B)	96	276	658	1115	473	99	0	7	5	13	451	2949	6142
Deletion	0	2	284	410	1	4	0	2	0	2	273	454	1432
Substitution	96	274	374	705	472	95	0	5	5	11	178	2495	4710
BA.2.75 (22D)	60	56	201	420	162	37	26	4	1	0	113	1032	2112
Deletion	0	0	84	98	0	0	0	2	0	0	84	83	351
Substitution	60	56	117	322	162	37	26	2	1	0	29	949	1761
BQ.1 (22E)	24	70	184	296	167	29	0	6	0	3	92	784	1655
Deletion	0	0	69	72	0	0	0	4	0	0	69	131	345
Substitution	24	70	115	224	167	29	0	2	0	3	23	653	1310
XBB (22F)	146	147	521	953	463	88	69	8	0	69	308	2836	5608
Deletion	0	0	219	228	0	2	0	1	0	2	219	284	955
stop codon	0	0	0	0	0	0	0	0	0	60	0	0	60
Substitution	146	147	302	725	463	86	69	7	0	7	89	2552	4593
XBB.1.5 (23A)	41	40	146	253	123	22	21	2	0	21	83	752	1504
Deletion	0	0	60	60	0	0	0	0	0	0	60	80	260
stop codon	0	0	0	0	0	0	0	0	0	20	0	0	20
Substitution	41	40	86	193	123	22	21	2	0	1	23	672	1224
XBB.1.16 (23B)	42	42	151	288	160	26	21	1	3	21	125	827	1707
Deletion	0	0	63	63	0	0	0	0	0	0	63	71	260

stop codon	0	0	0	0	0	0	0	0	0	21	0	0	21
Substitution	42	42	88	225	160	26	21	1	3	0	62	756	1426
CH.1.1 (23C)	20	19	71	163	78	11	12	0	0	1	40	349	764
Deletion	0	0	30	30	0	0	0	0	0	0	30	28	118
Substitution	20	19	41	133	78	11	12	0	0	1	10	321	646
XBB.1.9 (23D)	272	271	978	2015	909	151	128	8	2	141	685	5236	10796
Deletion	0	0	404	413	3	0	0	0	0	0	404	504	1728
stop codon	0	0	0	0	0	0	0	0	0	134	0	0	134
Substitution	272	271	574	1602	906	151	128	8	2	7	281	4732	8934
XBB.2.3 (23E)	34	36	125	240	102	18	17	19	0	4	71	724	1390
Deletion	0	0	51	51	0	0	0	0	0	0	51	93	246
Substitution	34	36	74	189	102	18	17	19	0	4	20	631	1144
Recombinant	7	18	45	81	37	10	1	0	0	1	24	199	423
Deletion	0	0	18	21	0	0	0	0	0	0	18	25	82
stop codon	0	0	0	0	0	0	0	0	0	1	0	0	1
Substitution	7	18	27	60	37	10	1	0	0	0	6	174	340

Table S4. Frequencies of the top 10 amino acid mutations in each protein.

**E**	**Freq**	**M**	**Freq**	**N**	**Freq**	**ORF1a**	**Freq**	**ORF1b**	**Freq**	**ORF3a**	**Freq**
E:T9I	866	M:Q19E	840	N:R203K	894	ORF1a:T3255I	987	ORF1b: P314L	1100	ORF3a: T223I	451
E:T11A	306	M:A63T	839	N:G204R	886	ORF1a:G3676-	909	ORF1b: I1566V	860	ORF3a: S26L	173
E:P71L	29	M:D3G	407	N:P13L	863	ORF1a:S3675-	909	ORF1b: G662S	478	ORF3a: K21N	39
E:A36-	24	M:I82T	173	N:E31-	860	ORF1a:P3395H	852	ORF1b: R1315C	461	ORF3a: S171L	32
E:L37-	21	M:D3N	115	N:R32-	860	ORF1a:F3677-	508	ORF1b: T2163I	461	ORF3a: Q57H	29
E:R38G	20	M:Q36-	27	N:S33-	858	ORF1a:S135R	460	ORF1b: S959P	246	ORF3a: L106F	15
E:V58F	4	M:L35-	12	N:S413R	449	ORF1a:L3027F	458	ORF1b: P1000L	168	ORF3a: L85F	12
E:F23S	3	M:F37-	10	N:D377Y	173	ORF1a:G1307S	455	ORF1b: A1918V	127	ORF3a: A54V	7
E:I33-	3	M:A38-	6	N:R203M	172	ORF1a:T842I	451	ORF1b: T1050N	68	ORF3a: D155Y	6
E:T30I	3	M:L34-	5	N:D63G	170	ORF1a:K856R	406	ORF1b: G1093S	33	ORF3a: L140F	6
**ORF6**	**Freq**	**ORF7a**	**Freq**	**ORF7b**	**Freq**	**ORF8**	**Freq**	**ORF9b**	**Freq**	**S**	**Freq**
ORF6:D61L	326	ORF7a:T120I	170	ORF7b:T40I	126	ORF8:G8*	237	ORF9b: E27-	860	S:D614G	1110
ORF6:F2S	23	ORF7a:V82A	165	ORF7b:E39*	12	ORF8:D119-	154	ORF9b: N28-	860	S:P681H	889
ORF6:S41F	3	ORF7a:A13V	17	ORF7b:L11F	3	ORF8:F120-	154	ORF9b: A29-	860	S:K417N	887
ORF6:F2-	2	ORF7a:L116F	8	ORF7b:A15S	2	ORF8:Q27*	28	ORF9b: P10S	836	S:H655Y	881
ORF6:I14M	2	ORF7a:T39I	4	ORF7b:H42Y	2	ORF8:R52I	26	ORF9b: T60A	170	S:N679K	863
ORF6:A12V	1	ORF7a:C113S	3	ORF7b:L32F	2	ORF8:Y73C	26	ORF9b: I5T	167	S:N440K	862
ORF6:E46V	1	ORF7a:G70C	3	ORF7b:L4F	2	ORF8:K68*	22	ORF9b: D16G	82	S:N969K	862
ORF6:F22L	1	ORF7a:L112-	3	ORF7b:S31L	2	ORF8:D35Y	10	ORF9b: P10F	28	S:Q954H	860
ORF6:F2L	1	ORF7a:T111-	3	ORF7b:A15T	1	ORF8:A65V	6	ORF9b: N55S	20	S:N764K	857
ORF6:I33T	1	ORF7a:T14I	3	ORF7b:A43S	1	ORF8:I121-	6	ORF9b: D89-	18	S:D796Y	847
